# Functional Roles of Long Non-coding RNAs in Motor Neuron Development and Disease

**DOI:** 10.1186/s12929-020-00628-z

**Published:** 2020-02-25

**Authors:** Kuan-Wei Chen, Jun-An Chen

**Affiliations:** Institute of Molecular Biology, Academia Sinica, Taipei, 11529 Taiwan

**Keywords:** Long non-coding RNA, Motor neuron, Spinal muscular atrophy, Amyotrophic lateral sclerosis

## Abstract

Long non-coding RNAs (lncRNAs) have gained increasing attention as they exhibit highly tissue- and cell-type specific expression patterns. LncRNAs are highly expressed in the central nervous system and their roles in the brain have been studied intensively in recent years, but their roles in the spinal motor neurons (MNs) are largely unexplored. Spinal MN development is controlled by precise expression of a gene regulatory network mediated spatiotemporally by transcription factors, representing an elegant paradigm for deciphering the roles of lncRNAs during development. Moreover, many MN-related neurodegenerative diseases, such as amyotrophic lateral sclerosis (ALS) and spinal muscular atrophy (SMA), are associated with RNA metabolism, yet the link between MN-related diseases and lncRNAs remains obscure. In this review, we summarize lncRNAs known to be involved in MN development and disease, and discuss their potential future therapeutic applications.

## Introduction

Next-generation RNA sequencing technology has revealed thousands of novel transcripts that possess no potential protein-coding elements. These RNAs are typically annotated as non-coding RNAs (ncRNAs) in the Human Genome Project and ENCODE Project [31, 59, 147]. Although most of the human genome is transcribed at certain stages during embryonic development, growth, or disease progression, ncRNAs were classically considered transcriptional noise or junk RNA due to their low expression levels relative to canonical mRNAs that generate proteins [19, 60]. However, emerging and accumulating biochemical and genetic evidences have gradually revealed their important regulatory roles in development and disease contexts [11, 109]. In principle, regulatory ncRNAs can be further divided into two groups depending on their lengths. Small RNAs are defined as being shorter than 200 nucleotides (nt), which include well-known small RNAs such as microRNA (miRNA, 22-25 nt), Piwi interacting RNA (piRNA, 21-35 nt), small nucleolar RNA (snoRNA, 60-170 nt), and transfer RNA (tRNA, 70-100 nt). NcRNAs longer than 200 nt are termed as long non-coding RNAs (lncRNAs) that comprise about 10~30% of transcripts in both human (GENCODE 32) and mouse (GENCODE M23) genomes, suggesting that they may play largely unexplored roles in mammal physiology. LncRNAs can be classified further according to their genomic location. They can be transcribed from introns (intronic lncRNA), coding exons, 3' or 5' untranslated regions (3' or 5' UTRs), or even in an antisense direction overlapping with their own transcripts (natural antisense transcript, NAT) [64, 130]. In regulatory regions, upstream of promoters (promoter upstream transcript, PROMPT) [106], enhancers (eRNA) [76], intergenic regions (lincRNA) [114] and telomeres [81] can be other sources of lncRNAs. Many hallmarks of lncRNA processing are similar to those of mRNAs in post-transcription, such as nascent lncRNAs being 5'-capped, 3'-polyadenylated or alternatively spliced [19]. LncRNA production is less efficient than for mRNAs and their half-lives appear to be shorter [98]. Unlike mRNA that is directly transported to the cytoplasm for translation, many lncRNAs tend to be located in the nucleus rather than in the cytosol, as revealed by experimental approaches such as fluorescent *in situ* hybridization [20, 67]. However, upon export to cytoplasm, some lncRNAs bind to ribosomes where they can be translated into functional peptides under specific cell contexts [20, 58]. For instance, myoregulin is encoded by a putative lncRNA and binds to sarco/endoplasmic reticulum Ca^2+^-ATPase (SRCA) to regulate Ca^2+^ import in the sarcoplasmic reticulum [6]. Nevertheless, it remains to be established if other ribosome-associated lncRNAs generate functional peptides.

### General function of lncRNAs

**Fig. 1:**
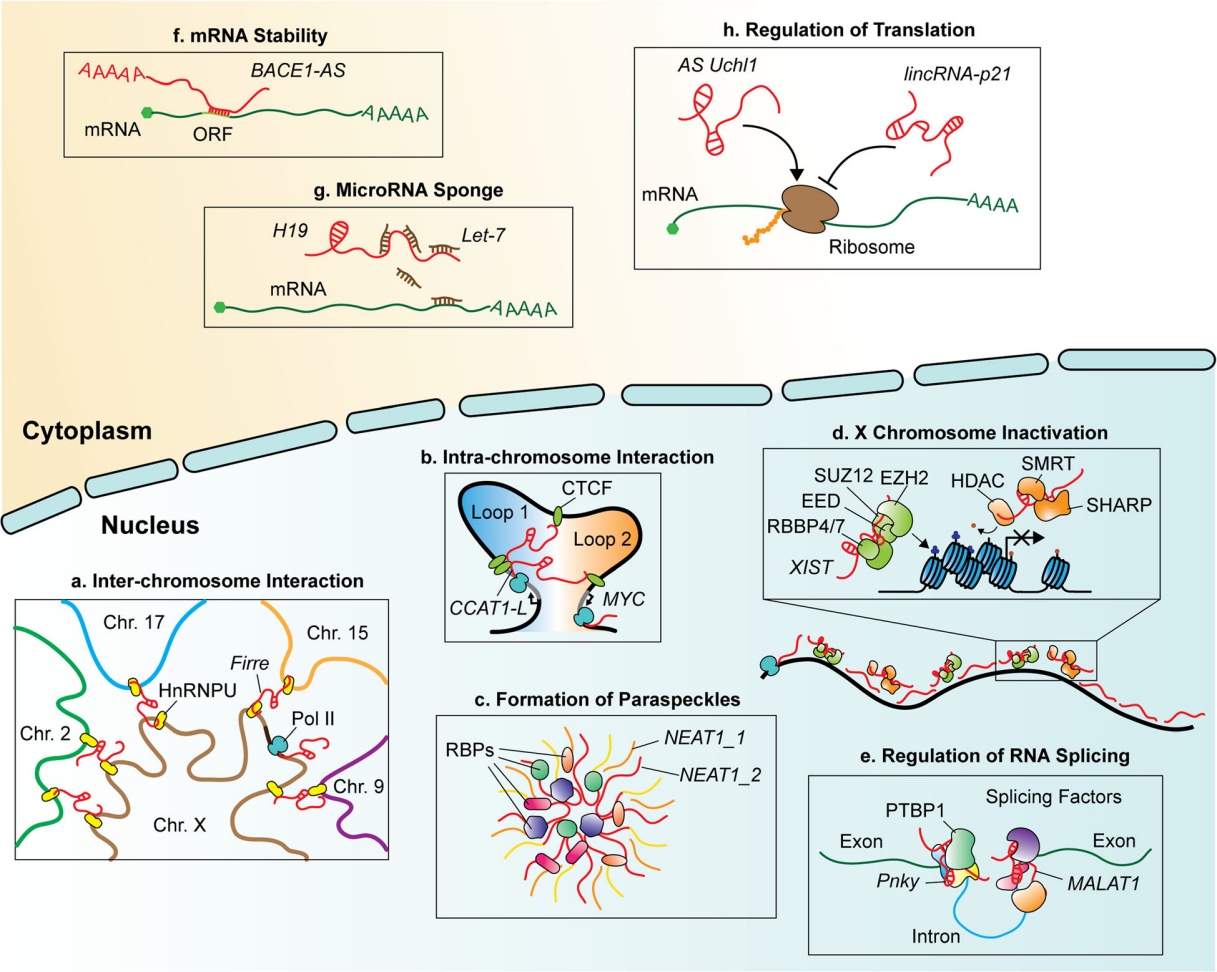
Summary (with examples) of the multifaceted roles of lncRNAs in the cell. **a** The X chromosome-derived lncRNA *Firre* associates with HnRNPU to establish inter-chromosome architecture. **b***CCAT1-L* generated from upstream of *MYC* loci promotes *MYC* expression via CTCF-mediated looping. **c** Paraspeckle formation is regulated by interactions between *NEAT1_2* and RBPs. **d** X chromosome inactivation is accomplished by coordination between *Xist*-PRC2-mediated deposition of H3K27me3 and *Xist*-SMRT/SHARP/HDAC-mediated deacetylation of H3ac. **e** Facilitation of RNA splicing by *Pnky*/PTBP1 and *Malat1*/RBPs complexes. **f***BACE1-AS* associates with *BACE1* mRNA via the open reading frame to stabilize *BACE1* mRNA. **g***H19* lncRNA sequesters *let-7* miRNA to prevent *let-7*-mediated gene suppression. **h** Antisense *Uchl1* promotes but *lincRNA-p21* inhibits the translation process.

A broad spectrum of evidence demonstrates the multifaceted roles of lncRNAs in regulating cellular processes. In the nucleus, lncRNAs participate in nearly all levels of gene regulation, from maintaining nuclear architecture to transcription *per se*. To establish nuclear architecture, *Functional intergenic repeating RNA element* (*Firre*) escapes from the X chromosome inactivation (XCI) and bridges multi-chromosomes, partly via association with heterogeneous nuclear ribonucleoprotein U (hnRNPU) (Figure 1a) [54]. CCCTC-binding factor (CTCF)-mediated chromosome looping can also be accomplished by lncRNAs. For example, *colorectal cancer associated transcript 1 long isoform* (*CCAT1-L*) facilitates promoter-enhancer looping at the *MYC* locus by interacting with CTCF, leading to stabilized *MYC* expression and tumorigenesis (Figure 1b) [153]. In addition, CTCF binds to many X chromosome-derived lncRNAs such as *X-inactivation intergenic transcription element* (*Xite*), *X-inactive specific transcript* (*Xist*) and the reverse transcript of *Xist* (*Tsix*) to establish three-dimensional organization of the X chromosome during XCI [69]. In addition to maintaining nuclear architecture, lncRNAs may also serve as building blocks of nuclear compartments. For example, *nuclear enriched abundant transcript 1* (*NEAT1*) is the core element of paraspeckles that participate in various biological processes such as nuclear retention of adenosine-to-inosine-edited mRNAs to restrict their cytoplasmic localization and viral infection response. However, the exact function of paraspeckles has yet to be fully deciphered (Figure 1c) [26, 30, 57]. LncRNAs can also function as a scaffolding component, bridging epigenetic modifiers to coordinate gene expression (e.g. activation or repression). For instance, *Xist* interacts with polycomb repressive complex 2 (PRC2) and the silencing mediator for retinoid and thyroid hormone receptor (SMRT)/histone deacetylase 1 (HDAC1)-associated repressor protein (SHARP) to deposit a methyl group on lysine residue 27 of histone H3 (H3K27) and to deacetylate histones, respectively, leading to transcriptional repression of the X chromosome (Figure 1d) [87]. Similarly, *Hox antisense intergenic RNA* (*Hotair*) bridges the PRC2 complex and lysine-specific histone demethylase 1A (LSD1, a H3K4me2 demethylase) to synergistically suppress gene expression [118, 140]. In contrast, *HOXA transcript at the distal tip* (*HOTTIP*) interacts with the tryptophan-aspartic acid repeat domain 5 - mixed-lineage leukemia 1 (WDR5-MLL1) complex to maintain the active state of the 5' *HOXA* locus via deposition of histone 3 lysine 4 tri-methylation (H3K4me3) [149]. LncRNAs also regulate the splicing process by associating with splicing complexes. A neural-specific lncRNA, *Pnky*, associates with the splicing regulator polypyrimidine tract-binding protein 1 (PTBP1) to regulate splicing of a subset of neural genes [112]. Moreover, interaction between *Metastasis-associated lung adenocarcinoma transcript 1* (*Malat1*) and splicing factors such as serine/arginine rich splicing factor 1 (SRSF1) is required for alternative splicing of certain mRNAs (Figure 1e) [139].

Apart from nucleus, lncRNAs in the cytoplasm are typically involved in mRNA biogenesis. For example, in Alzheimer’s disease (AD), *β-secretase-1 antisense RNA* (*BACE1-AS*) derived within an important AD-associated enzyme, *BACE1*, elevates BACE1 protein levels by stabilizing its mRNA through a post-translational feed-forward loop [44]. Mechanistically, *BACE1-AS* masks the miRNA-485-5p binding site at the open reading frame of *BACE1* mRNA to maintain *BACE1* mRNA stability (Figure 1f) [45]. *H19*, a known imprinting gene expressed as a lncRNA from the maternal allele, promotes myogenesis by sequestering *lethal-7* (*let-7*) miRNAs that, in turn, prevents *let-7*-mediated gene repression (Figure 1g) [62]. LncRNAs not only regulate transcription but also affect translation. Human *lincRNA-p21* (Trp53cor1) disrupts translation of *CTNNB1* and *JUNB* via base-pairing at multiple sites of the 5' and 3' UTR and coding regions, resulting in recruitment of the translational repressors RCK and fragile X mental retardation protein (FMRP) to suppress translation (Figure 1h, right) [158]. In contrast, an antisense RNA generated from *ubiquitin carboxyterminal hydrolase L1* (*AS Uchl1*) promotes translational expression of Uchl1 protein via its embedded short interspersed nuclear elements B2 (SINEB2). In the same study, inhibition of mammalian target of rapamycin complex 1 (mTORC1) was shown to trigger cytoplasmic localization of *AS Uchl1* and to increase the association between polysomes and *Uchl1* mRNA in a eukaryotic translation initiation factor 4F (eIF4F) complex independently of translation (Figure 1h, left) [21]. Finally, compared to mRNAs, lncRNAs seem to manifest a more tissue-specific manner [19]. In agreement with this concept, genome-wide studies have revealed that large numbers of tissue-specific lncRNAs are enriched in brain regions and some of them are involved in neurogenesis [7, 15, 37, 89]. We discuss some of these lncRNAs in greater detail below, with a particular focus on their roles during spinal MN development as this latter serves as one of the best paradigms for studying the development and degeneration of the central nervous system (CNS).

#### Role of lncRNAs in regulating neural progenitors

**Fig. 2:**
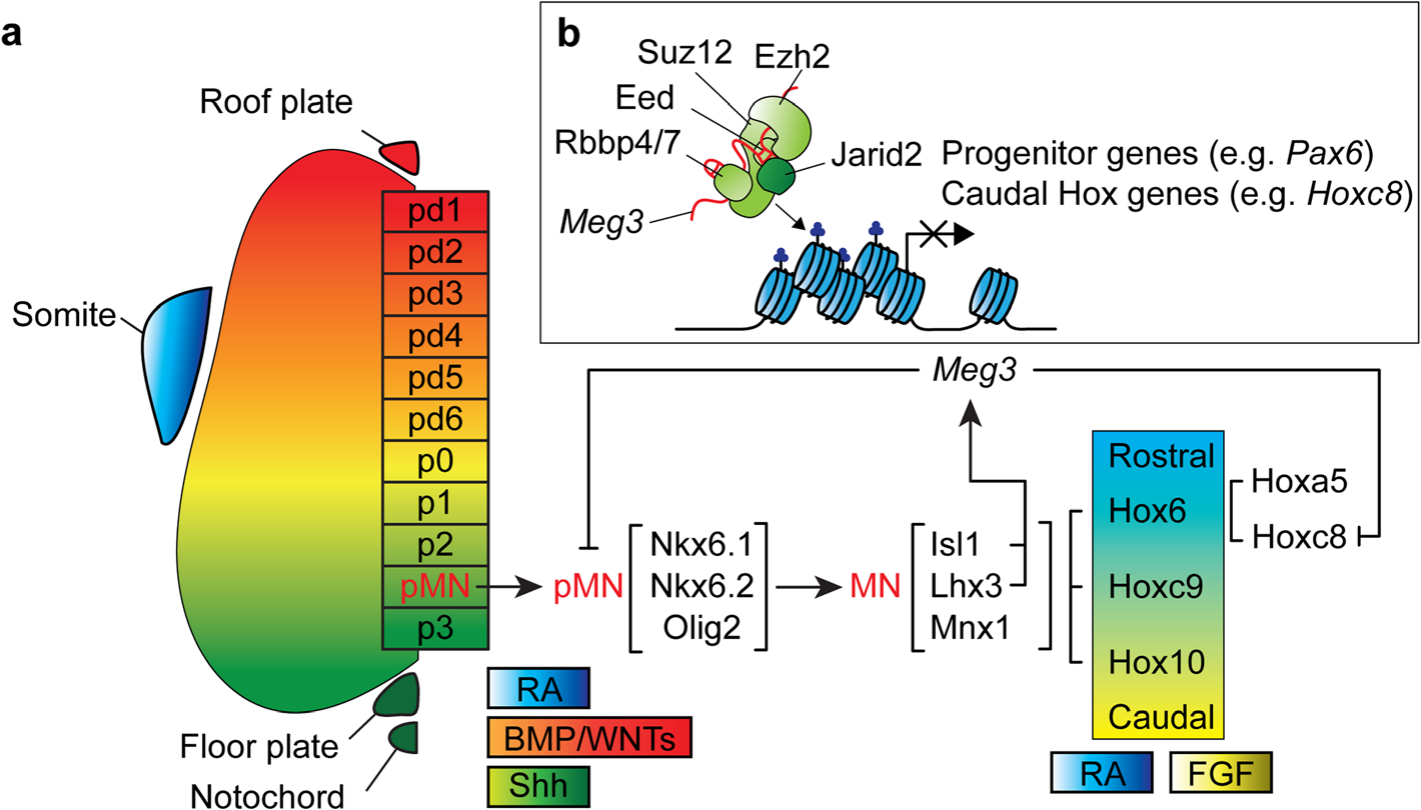
Schematic illustration of spinal motor neuron development. **a** Notochord- and floor plate-derived sonic hedgehog protein (Shh), and roof plate-generated wingless/integrated (WNT) protein and bone morphogenetic (BMP) protein, as well as retinoic acid (RA) diffusing from the paraxial mesoderm, pattern the identities of spinal neurons by inducing cross-repressive transcription factors along the dorso-ventral axis (pd1~6, p0, p1, p2, pMN, and p3). Motor neuron progenitors (pMNs) are generated by co-expression of Olig2, Nkx6.1 and Nkx6.2. After cell cycle exit, pMNs give rise to generic MNs by concomitantly expressing Isl1, Lhx3 and Mnx1. Along the rostro-caudal axis, Hox6/Hoxc9/Hox10 respond to RA and fibroblast growth factor (FGF) to pattern the brachial, thoracic and lumbar segments, respectively. **b** In the Hox6^on^ segment, the interaction between PRC2-Jarid2 complex and a Isl1/Lhx3 induced lncRNA *Meg3* perpetuates the brachial Hoxa5^on^ MN by repressing caudal Hoxc8 and alternative progenitor genes Irx3 and Pax6 via the maintenance of H3K27me3 epigenetic landscape in these genes. Yet the detailed mechanism how *Meg3* targets to these selective genes still needs to be illustrated.

As part of the CNS, spinal MNs are located in the ventral horn of the spinal cord that conveys signals from the brainstem or sensory inputs to the terminal muscles, thereby controlling body movements. MN development requires precise spatiotemporal expression of extrinsic and intrinsic factors. Upon neurulation, the wingless/integrated protein family (WNT) and the bone morphogenetic protein family (BMP) are secreted from the roof plate of the developing neural tube to generate a dorsal to ventral gradient [4, 88]. In contrast, sonic hedgehog (Shh) proteins emanating from the floor plate as well as the notochord generate an opposing ventral to dorsal gradient [16]. Together with paraxial mesoderm-expressed retinoic acid (RA), these factors precisely pattern the neural tube into spinal cord progenitor domains pd1~6, p0, p1, p2, motor neuron progenitor (pMN), and p3 along the dorso-ventral axis (Figure 2a). This patterning is mediated by distinct expression of cross-repressive transcription factors—specifically, Shh-induced class II transcription factors (Nkx2.2, Nkx2.9, Nkx6.1, Nkx6.2, Olig2) or Shh-inhibited class I transcription factors (Pax3, Pax6, Pax7, Irx3, Dbx1, Dbx2)—that further define the formation of each progenitor domain [104, 143]. All spinal MNs are generated from pMNs, and pMNs are established upon co-expression of Olig2, Nkx6.1 and Nkx6.2 under conditions of high Shh levels [2, 105, 132, 162]. Although a series of miRNAs have been shown to facilitate patterning of the neuronal progenitors in the spinal cord and controlling of MN differentiation [24, 25, 27, 74, 141, 142], the roles of lncRNAs during MN development are just beginning to emerge. In Table 1, we summarize the importance of lncRNAs for the regulation of transcription factors in MN contexts. For instance, the lncRNA *lncrps25* is located near the *S25* gene (which encodes a ribosomal protein) and it shares high sequence similarity with the 3' UTR of neuronal regeneration-related protein (NREP) in zebrafish. Loss of *lncrps25* reduces locomotion behavior by regulating pMN development and Olig2 expression [48]. Additionally, depletion of an MN-enriched lncRNA, i.e. *Maternally expressed gene 3* (*Meg3*), results in upregulation of progenitor genes (i.e., *Pax6* and *Dbx1*) in embryonic stem cell (ESC)-derived post-mitotic MNs, as well as in post-mitotic neurons in embryos. Mechanistically, *Meg3* associates with the PRC2 complex to facilitate the maintenance of H3K27me3 levels in many progenitor loci, including *Pax6* and *Dbx1* (Figure 2b) [156]. Apart from lncRNA-mediated regulation of *Pax6* in the spinal cord, corticogenesis in primates also seems to rely on the Pax6/lncRNA axis [113, 145]. In this scenario, primate-specific *lncRNA neuro-development* (*Lnc-ND*) located in the 2p25.3 locus [131] exhibits an enriched expression pattern in neuronal progenitor cells but reduced expression in the differentiated neurons. Microdeletion of the 2p25.3 locus is associated with intellectual disability. Manipulations of *Lnc-ND* levels reveals that *Lnc-ND* is required for Pax6 expression and that overexpression of *Lnc-ND* by means of *in utero* electroporation in mouse brain promotes expansion of the Pax6-positive radial glia population [113]. Moreover, expression of the *Neurogenin 1* (*Ngn1*) upstream enhancer-derived eRNA, *utNgn1*, is necessary for expression of *Ngn1* itself in neocortical neural precursor cells and it is suppressed by PcG protein at the ESC stage [108]. Thus, lncRNAs seem to mediate a battery of transcription factors that are important for early neural progenitor patterning and this role might be conserved across vertebrates.

**Table 1:** Proposed functions of lncRNAs during spinal motor neuron development

LncRNA	Proposed function	Organism/cell models	Reference
*Lncrps25*	Affects Olig2 expression	Zebrafish	[48]
*CAT7/cat7l*	Recruitment of PRC1 and PRC2 complexes to suppress *MNX1* expression in progenitor MNs	Zebrafish/hESC~MNs	[115]
*Meg3*	Association with PRC2 complex to repress MN progenitor and caudal *Hox* genes in cervical MNs	Mouse/mESC~MNs	[156]

#### LncRNAs in the regulation of postmitotic neurons

In addition to their prominent functions in neural progenitors, lncRNAs also play important roles in differentiated neurons. Taking spinal MNs as an example, postmitotic MNs are generated from pMNs, and after cell cycle exit they begin to express a cohort of MN-specific markers such as Insulin gene enhancer protein 1 (Isl1), LIM/homeobox protein 3 (Lhx3), and Motor neuron and pancreas homeobox 1 (Mnx1, Hb9) (Figure 2a). Isl1/Lhx3/NLI forms an MN-hexamer complex to induce a series of MN-specific regulators and to maintain the terminal MN state by repressing alternative interneuron genes [43, 72]. Although the gene regulatory network for MN differentiation is very well characterized, the role of the lncRNAs involved in this process is surprisingly unclear. Only a few examples of that role have been uncovered. For instance, the lncRNA *CAT7* is a polyadenylated lncRNA that lies upstream (~400 kb) of *MNX1* identified from the RNA-Polycomb repressive complex 1 (PRC1) interactome. Loss of *CAT7* results in de-repression of *MNX1* before committing to neuronal lineage through reduced PRC1 and PRC2 occupancy at the *MNX1* locus in hESC~MNs [115]. Furthermore, an antisense lncRNA (*MNX1-AS1*) shares the same promoter as MNX1, as revealed by clustered regularly interspaced short palindromic repeats (CRISPR) and CRISPR-associated protein 9 (CRISPR-Cas9) screening [53]. These results suggest that in addition to neural progenitors, lncRNAs could have another regulatory role in fine-tuning neurogenesis upon differentiation. However, whether the expression and functions of these lncRNAs are important for MN development *in vivo* still needs to be further validated. Future experiments to systematically identify lncRNAs involved in this process will greatly enhance our knowledge about lncRNAs and their mysterious roles in early neurogenesis.

After generic postmitotic MNs have been produced, they are further programmed into versatile subtype identities along the rostro-caudal spinal cord according to discrete expression of signaling molecules, including retinoic acid (RA), WNT, fibroblast growth factor (FGF), and growth differentiation factor 11 (GDF11), all distributed asymmetrically along the rostro-caudal axis (Figure 2a). Antagonistic signaling of rostral RA and caudal FGF/GDF11 further elicits a set of Homeobox (Hox) proteins that abut each other, namely Hox6, Hox9 and Hox10 at the brachial, thoracic and lumbar segments, respectively [12, 77, 129]. These Hox proteins further activate downstream transcription factors that are required to establish MN subtype identity. For instance, formation of lateral motor column (LMC) MNs in the brachial and lumbar regions is regulated by Hox-activated Forkhead box protein P1 (Foxp1) [35, 119]. It is conceivable that lncRNAs might also participate in this MN subtype diversification process. For example, the lncRNA *FOXP1-IT1*, which is transcribed from an intron of the human *FOXP1* gene, counteracts integrin Mac-1-mediated downregulation of FOXP1 partly by decoying HDAC4 away from the *FOXP1* promoter during macrophage differentiation [128]. However, it remains to be verified if this Foxp1/lncRNA axis is also functionally important in a spinal cord context. An array of studies in various cell models has demonstrated regulation of *Hox* genes by lncRNAs such as *Hotair*, *Hottip* and *Haglr* [118, 149, 160]. However, to date, only one study has established a link between the roles of lncRNAs in MN development and *Hox* regulation. Using an embryonic stem cell differentiation system, a battery of MN hallmark lncRNAs have been identified [14, 156]. Among these MN-hallmark lncRNAs, knockdown of *Meg3* leads to the dysregulation of *Hox* genes whereby caudal *Hox* gene expression (*Hox9*~*Hox13*) is increased but rostral *Hox* gene expression (*Hox1*~ *Hox8*) declines in cervical MNs. Analysis of maternally-inherited intergenic differentially methylated region deletion (*IG-DMR*^*matΔ*^) mice in which *Meg3* and its downstream transcripts are further depleted has further revealed ectopic expression of caudal Hoxc8 in the rostral Hoxa5 region of the brachial segment, together with a concomitant erosion of Hox-mediated downstream genes and axon arborization (Figure 2b) [156]. Given that dozens of lncRNAs have been identified as hallmarks of postmitotic MNs, it remains to be determined if these other lncRNAs are functionally important *in vivo*. Furthermore, lncRNA knockout has been shown to exert a very mild or no phenotype *in vivo* [52]. Based on several lncRNA-knockout mouse models, it seems that the physiological functions of lncRNAs might not be as prominent as transcription factors during the developmental process [8, 123], yet their functions become more critical under stress conditions such as cancer progression or neurodegeneration [102, 124]. Therefore, next we discuss how lncRNAs have been implicated in MN-related diseases.

#### Motor neuron-related diseases

Since lncRNAs regulate MN development and function, it is conceivable that their dysregulation or mutation would cause neurological disorders. Indeed, genome-wide association studies (GWAS) and comparative transcriptomic studies have associated lncRNAs with a series of neurodegenerative diseases, including the age-onset MN-associated disease amyotrophic lateral sclerosis (ALS) [86, 164]. Similarly, lncRNAs have also been linked to spinal muscular atrophy (SMA) [33, 152]. However, most of these studies have described associations but do not present unequivocal evidence of causation. Below and in Table 2, we summarize some of these studies linking lncRNAs to MN-related diseases.

**Table 2:** Proposed functions of lncRNAs in spinal motor neuron diseases

LncRNA	Disease	Proposed function	Organism/cell models	Reference
*ATXN2-AS*	ALS	(CUG)_n_ repeat expansions induce neurotoxicity.	SK-N-MC neuroblastoma cells and lymphoblastoid cell lines from ALS patients	[75]
*C9ORF72 antisense RNA*	ALS	Forms RNA foci and repeat-associated non-AUG (RAN) translation generates dipeptides to cause neurotoxicity.	Drosophila, Zebrafish/Neuro-2a, mouse primary cortical and motor neurons	[91, 96, 134, 138, 151, 155, 161]
*NEAT1*	ALS	Facilitates paraspeckle formation. High levels of *NEAT1* trigger neurotoxicity.	Mouse/NSC-34 MN-like cells	[30, 133]
*SMN-AS1*	SMA	Recruits PRC2 complex to suppress the *SMN* gene.	Mouse/human SMA-iPSC-derived MNs, *SMNΔ7* mouse cortical neurons	[33, 152]

#### Amyotrophic lateral sclerosis (ALS)

ALS is a neurodegenerative disease resulting in progressive loss of upper and lower MNs, leading to only 5-10 years median survival after diagnosis. More than 90% of ALS patients are characterized as sporadic (sALS), with less than 10% being diagnosed as familial (fALS) [17]. Some ALS-causing genes—such as *superoxide dismutase 1* (*SOD1*) and *fused in sarcoma/translocated in sarcoma* (*FUS/TLS*)—have been identified in both sALS and fALS patients, whereas other culprit genes are either predominantly sALS-associated (e.g. unc-13 homolog A, UNC13A) or fALS-associated (e.g. D-amino acid oxidase, DAO). These findings indicate that complex underlying mechanisms contribute to the selective susceptibility to MN degeneration in ALS. Since many characterized ALS-causing genes encode RNA-binding proteins (RBPs)—such as angiogenin (ANG), TAR DNA-binding protein 43 (TDP-43), FUS, Ataxin-2 (ATXN2), chromosome 9 open reading frame 72 (C9ORF72), TATA-box binding protein associated factor 15 (TAF15) and heterogeneous nuclear ribonucleoprotein A1 (HNRNPA1)—it is not surprising that global and/or selective RBP-RNAs, including lncRNAs, might participate in ALS onset or disease progression. Below, we discuss some representative examples.

### Nuclear Enriched Abundant Transcript 1 (NEAT1)

*NEAT1* is an lncRNA that appears to play an important structural role in nuclear paraspeckles [30]. Specifically, there are two *NEAT1* transcripts: *NEAT1_1* (3.7 kb) is dispensable whereas *NEAT1_2* (23 kb) is essential for paraspeckle formation [30, 100]. However, expression of *NEAT1_2* is low in the CNS of mouse ALS models relative to ALS patients, indicating a difference between rodent and human systems [101, 103]. Although crosslinking and immunoprecipitation assay (CLIP) has revealed that *NEAT1* associates with TDP-43 [103, 137, 154] and FUS/TLS [103], the first evidence linking *NEAT1* and paraspeckles to ALS was the observation of co-localization of *NEAT1_2* with TDP-43 and FUS/TLS in paraspeckles of early-onset ALS patients [103]. A more detailed analysis has revealed that *NEAT1_2* is highly enriched in neurons of the anterior horn of the spinal cord and in cortical tissues of ALS patients [126, 137]. Indeed, increased paraspeckle formation has been reported in the spinal cords of sALS and fALS patients relative to healthy individuals [126], indicating that paraspeckle formation might be a common hallmark of ALS patients. Interestingly, by utilizing an ESC-derived neuron system, a significant increase in paraspeckles was observed at the neuron progenitor stage, suggesting that paraspeckles may exist in the short time-window of neural development [126]. Manipulating ALS-related RBPs (i.e. FUS, TDP-43, and MATR3) impacts levels of *NEAT1*, showing that these RBPs not only interact with *NEAT1* but also regulate *NEAT1* RNA levels. The level of *NEAT1_2* increases upon FUS, TDP-43 or MATR3 deletion [10, 100]. In contrast, elimination of TAF15, hnRNPA1 or splicing factor proline and glutamine rich (SFPQ) downregulates *NEAT1_2* levels [103]. There are conflicting results with regard to whether manipulation of TDP-43 affects *NEAT1_2* [100, 126]. Introducing patient-mutated FUS (e.g. P525L) also results in impaired paraspeckle formation by regulating *NEAT1* transcription and misassemble of other paraspeckle proteins in the cytoplasm or nucleus [5, 127]. Together, these results seem to indicate that mutation of ALS-related RBPs affects *NEAT1* expression and paraspeckle formation during disease progression.

Although many studies have depicted how mutated ALS-related proteins regulate paraspeckle formation, levels of *NEAT1_2*, inappropriate protein assembly into granules or sub-organelles, and the role of *NEAT1_2* in ALS progression remain poorly understood. Recently, direct activation of endogenous *NEAT1* using a CRISPR-Cas9 system suggested that elevated *NEAT1* expression is somewhat neurotoxic in NSC-34 cells, a mouse MN-like hybrid cell line. Though no direct evidence showing that this effect is mediated by *NEAT1_2* was presented in that study, it did at least exclude *NEAT1_1* as the mediator [133]. This outcome may imply that increased *NEAT1_2* facilitates paraspeckle formation and also somehow induces cell death or degeneration. However, more direct evidence of correlations and concordant links between RBP-lncRNA associations and ALS are needed to strengthen the rationale of utilizing lncRNAs for future therapeutic purposes.

### C9ORF72 antisense RNA

In 2011, the *C9ORF72* gene with a hexanucleotide GGGGCC (G_4_C_2_) repeat expansion was identified as the most frequent genetic cause of both ALS and frontotemporal dementia (FTD) in Europe and North America [36, 117]. ALS and FTD represent a disease spectrum of overlapping genetic causes, with some patients manifesting symptoms of both diseases. Whereas ALS is defined by loss of upper and/or lower MNs leading to paralysis, FTD is characterized by degeneration of the frontal and temporal lobes and corresponding behavioral changes. The abnormal (G_4_C_2_) repeat expansion in the first intron of *C9ORF72* not only accounts for almost 40% of fALS and familial FTD (fFTD), but it is also found in a small cohort of sALS and sporadic FTD (sFTD) patients [36, 85, 111, 117]. Healthy individuals exhibit up to 20 copies of the (G_4_C_2_) repeat, but it is dramatically increased to hundreds to thousands of copies in ALS patients [36]. Loss of normal C9ORF92 protein function and gain of toxicity through abnormal repeat expansion have both been implicated in *C9ORF72*-associated FTD/ALS. Several *C9ORF72* transcripts have been characterized and, surprisingly, antisense transcripts were found to be transcribed from intron 1 of the *C9ORF72* gene [97]. Both *C9ORF72* sense (*C9ORF72-S*) and antisense (*C9ORF72-AS*) transcripts harboring hexanucleotide expansions could be translated into poly-dipeptides and were found in the MNs of *C9ORF72*-associated ALS patients [47, 50, 95, 121, 151, 163]. Although *C9ORF72-S* RNA and consequent proteins have been investigated extensively, the functional relevance of *C9ORF7-AS* is still poorly understood. *C9ORF72-AS* contains the reverse-repeated hexanucleotide (GGCCCC, G_2_C_4_) located in intron 1. Similar to *C9ORF72-S*, *C9ORF72-AS* also forms RNA foci in brain regions such as the frontal cortex and cerebellum, as well as the spinal cord (in MNs and occasionally in interneurons) of ALS [49, 163] and FTD patients [36, 49, 92]. Intriguingly, a higher frequency of *C9ORF72-AS* RNA foci and dipeptides relative to those of *C9ORF72-S* have been observed in the MNs of a *C9ORF72*-associated ALS patient, with a concomitant loss of nuclear TDP-43 [32]. In contrast, another study suggested that compared to *C9ORF72-S*-generated dipeptides (poly-Gly-Ala and poly-Gly-Arg), fewer dipeptides (poly-Pro-Arg and poly-Pro-Ala) derived from *C9ORF72-AS* were found in the CNS region of *C9ORF72*-associated FTD patients [83]. These apparently contradictory results perhaps are due to differing sensitivities of the antibodies used in those studies. It has further been suggested that a fraction of the *C9ORF72-AS* RNA foci is found in the perinucleolar region, indicating that nucleolar stress may contribute to *C9ORF72*-associated ALS/FTD disease progression [70, 93, 136]. Interestingly, compared to the *C9ORF72-S* G_4_C_2_ repeats, a large number of *C9ORF72-AS* G_2_C_4_ repeats are associated with mono-ribosomes [135], suggesting that fewer dipeptides are generated in the former scenario. This outcome may indicate that *C9ORF72-AS* RNA may also contribute to the pathology caused by C9ORF72 hexanucleotide repeat expansion. Whereas *C9ORF72-S* can form G-quadruplexes [46, 55, 116] that are known to regulate transcription and gene expression [150], the C-rich *C9ORF72-AS* repeats may not form similar structures. Instead, the G_2_C_4_ expansions in *C9ORF72-AS* may form a C-rich motif [65] that likely affects genome stability and transcription [1]. Notably, an A-form-like double-helix with a tandem C:C mismatch has been observed in a crystal structure of the *C9ORF72-AS* repeat expansion, suggesting that different structural forms of *C9ORF72-AS* might regulate disease progression [38]. Thus, during disease progression, not only may *C9ORF72-AS* form RNA foci to sequester RBPs, but it could also indirectly regulate gene expression via its secondary structure.

Several C9ORF72 gain-of-function and loss-of-function animal models have been generated [9, 91, 138, 155]. A new *Drosophila melanogaster* (fly) model expressing the G_4_C_2_ or G_2_C_4_ RNA repeat followed by polyA (termed “polyA”) or these repeats within spliced GFP exons followed by polyA (termed “intronic”) reveals that both sense and antisense “polyA” accumulates in cytoplasm but sense and antisense “intronic” occur in the nucleus, with this latter mimicking actual pathological conditions [94]. However, expression of these repeated RNAs does not result in an obvious motor deficit phenotype, such as climbing ability of the *Drosophila* model, indicating that the repeats *per se* may not be sufficient to induce disease progression [94]. Nevertheless, applying that approach in a *Danio rerio* (zebrafish) model resulted in an outcome contradictory to that in *Drosophila*, with both sense and antisense repeated RNAs inducing clear neurotoxicity [134]. This discrepancy may be due to differing tolerances to RNA toxicity between the model species and the status of their neurons. Several mouse models have been established by introducing human *C9ORF72* repeats only or the gene itself with its upstream and downstream regions via transduction of adeno-associated virus (AAV) or bacterial artificial chromosome (BAC) constructs (reviewed in [9]). In the models that harbor full-length human *C9ORF72* with repeat expansions as well as upstream and downstream regions, dipeptide inclusions and RNA foci from *C9ORF72-S* and *-AS* have been observed and some of them develop motor [78] or cognition (working and spatial memory) defects [61] but others appear normal [107, 110]. Similarly, utilizing differentiated MNs from patient-derived induced pluripotent stem cells (iPSCs), *C9ORF72*-associated dipeptides and RNA foci have been observed but some of the expected pathologies were not fully recapitulated [3, 34, 39, 80]. These inconsistent findings may be due to the different genetic backgrounds used or the differing stress conditions applied.

Most studies on C9ORF72 have focused on the pathology caused by repeat expansion, but how C9ORF72 itself is regulated is only beginning to be revealed. Knockdown of a transcription elongation factor, Spt4, rescues C9ORF72-mediated pathology in a *Drosophila* model and decreases *C9ORF72-S* and *-AS* transcripts as well as poly-Gly-Pro protein production in iPSC-derived neurons from a *C9ORF72*-associated ALS patient [66]. Another CDC73/PAF1 protein complex (PAF1C), which is a transcriptional regulator of RNA polymerase II, has been shown to positively regulate both *C9ORF72-S* and *-AS* repeat transcripts [51]. Moreover, reduced expression of hnRNPA3, an G_4_C_2_ repeat RNA binding protein, elevates the G_4_C_2_ repeat RNA and dipeptide production in primary neurons [96]. Nevertheless, the RNA helicase DDX3X mitigates pathologies elicited by C9ORF72 repeat expansion by binding to G_4_C_2_ repeat RNA, which in turn inhibits repeat-associated non-AUG translation (RAN) but does not affect antisense G_2_C_4_ repeat RNA in iPSC-derived neurons and the *Drosophila* model [28]. Collectively, these findings reveal an alternative strategy for targeting C9ORF72 repeat expansions in that antisense oligonucleotides (ASOs) could be utilized against *C9ORF72-S* to attenuate RNA foci and reverse disease-specific transcriptional changes in iPSC-derived neurons [39, 122, 161].

### Ataxin 2 antisense (ATXN2-AS) transcripts

Ataxin-2 is an RBP and it serves as a genetic determinant or risk factor for various diseases including spinocerebellar ataxia type II (SCA2) and ALS. *ATXN2-AS* is transcribed from the reverse strand of intron 1 of the *ATXN2* gene. Similar to the G_4_C_2_ repeats of *C9ORF72-AS*, the (CUG)_n_ expansions of *ATXN2-AS* may promote mRNA stability by binding to U-rich motifs in mRNAs and they have been associated with ALS risk [40, 157]. Furthermore, *ATXN2-AS* with repeat expansions were shown to induce neurotoxicity in cortical neurons in a length-dependent manner [75]. In that same study, the authors also demonstrated that it is the transcripts rather than the polypeptides generated via RAN translation that are responsible for neurotoxicity. It has been suggested that the toxicity of CUG repeats is due to hairpin formation sequestering RBPs in the cell [68]. Thus, it is likely that the RNA repeats of *ATXN2-AS* or *C9ORF72-S/AS* might function in parallel to RAN peptide-induced neurotoxicity to exacerbate degeneration of MNs in ALS.

### Other lncRNAs implicated in ALS

By means of an ESC~MN system, several lncRNAs have been shown to be dysregulated in loss-of-function FUS MNs. Compared to FUS^+/+^ MNs, *Lhx1os* upregulation and *lncMN-1* (*2610316D01Rik*) and *lncMN-2* (*5330434G04Rik*) downregulation were observed in FUS^P517L/P517L^ and FUS^-/-^ MNs, suggesting that loss of FUS function affects some lncRNAs conserved among mouse and human [14]. A series of lncRNAs that have not been directly implicated in ALS-associated genetic mutations have been identified to participate in ALS contexts. For instance, *MALAT1* that contributes to nuclear speckles formation exhibits increased expression and TDP-43 binding in the cortical tissues of sporadic frontotemporal lobar degeneration (FTLD) patients, whereas downregulation of *Meg3* is associated with expression and binding to TDP-43 in the same system [137]. UV-CLIP analysis has revealed that TDP-43 associates with other lncRNAs such as *BDNFOS* and *TFEBα* in SHSY5Y cells [154]. In muscle cells, *Myolinc* (*AK142388*) associates with TDP-43 to facilitate binding of this latter protein to myogenic genes, thereby promoting myogenesis [90]. However, whether these lncRNAs play roles in ALS progression needs to be further investigated.

Several studies using *Drosophila* as a model have uncovered relationships between lncRNAs and ALS. Knockdown of *CR18854*, an lncRNA associated with the RBP Staufen [71], rescues the climbing ability defects arising from dysregulated *Cabeza* (the orthologue of human FUS*,* hereafter referred to as dFUS) in *Drosophila* [99]. In contrast, knockdown of the lncRNA *heat shock RNA ω* (*hsrω*) in *Drosophila* MNs gives rise to severe motor deficiency by affecting presynaptic terminals. Mechanistically, *hsrω* interacts with dFUS, and depletion of *hsrω* results in dFUS translocation into the cytoplasm and abrogation of its nuclear function [79]. Levels of *hsrω* are positively regulated by TDP-43 via direct binding of TDP-43 to the *hsrω* locus in *Drosophila* [29]. The human orthologue of Drosophila *hsrω*, stress-induced *Satellite III repeat RNA* (*Sat III*), has also been shown to be elevated upon TDP-43 overexpression in the frontal cortex of FTLD-TDP patients [29]. It would be interesting to investigate the relationship between *Sat III* and ALS in human patients.

#### Spinal muscular atrophy (SMA)

Spinal muscular atrophy (SMA) is a genetic disorder characterized by prominent weakness and wasting (atrophy) of skeletal muscles due to progressive MN degeneration. SMA is the number one worldwide case of neurodegeneration-associated mortality in infants younger than two years old. SMA is caused by autosomal recessive mutation or deletion of the *Survival Motor Neuron 1* (*SMN1*) gene, which can be ameliorated by elevated expression of *SMN2*, a nearly identical paralogous gene of *SMN1* [82]. Since the discovery of SMN1-causing phenotypes in SMA two decades ago [73], many researchers have highlighted SMN2 regulation as a rational approach to boost the generation of full-length SMN2 to offset disease effects [18, 22]. Recently, accumulating evidence has shown a critical role for lncRNAs in regulating the expression of SMN protein. For example, the antisense lncRNA *SMN-AS1* derived from the *SMN* locus suppresses SMN expression, and species-specific non-overlapping SMN-antisense RNAs have been identified in mouse and human [33, 152]. In both these studies, *SMN-AS1* recruits the PRC2 complex to suppress expression of SMN protein, which could be rescued by either inhibiting PRC2 activity or by targeted degradation of *SMN-AS1* using ASOs. Moreover, a cocktail treatment of *SMN2* splice-switching oligonucleotides (SSOs), which enhanced inclusion of exon 7 to generate functional SMN2, with *SMN-AS1* ASOs enhanced mean survival of SMA mice from 18 days to 37 days, with ~25% of the mice surviving more than 120 days [33]. These finding suggest that in addition to SSO treatment, targeting *SMN-AS1* could be another potential therapeutic strategy for SMA. Moreover, transcriptome analysis has revealed certain lncRNA defects in SMA mice exhibiting early or late-symptomatic stages [13]. By comparing the translatomes (RNA-ribosome complex) of control and SMA mice, some of the lncRNAs were shown to bind to polyribosomes and to alter translation efficiency [13]. Although lncRNAs can associate with ribosomes and some of them generate functional small peptides, it needs to be established if this information is relevant in SMA contexts.

#### LncRNAs in liquid-liquid phase separation (LLPS) and motor neuron diseases

An emerging theme of many of the genetic mutations leading to the neurodegenerative MN diseases discussed above is their link to RBPs. Interestingly, many of these RBPs participate in granule formation and are associated with proteins/RNAs that undergo liquid-liquid phase separation (LLPS) (reviewed in [120]). LLPS is a phenomenon where mixtures of two or more components self-segregate into distinct liquid phases (e.g. separation of oil and water phases) and it appears to underlie formation of many transient membrane organelles, such as stress granules that contain many ribonucleoproteins (RNPs). Although it remains unclear why ubiquitously expressed RNP granule proteins aggregate in neurodegenerative disease, one study found that aggregated forms of mutant SOD1, a protein associated with fALS, accumulates in stress granules [41]. These aggregated forms induce mis-localization of several proteins associated with the miRNA biogenesis machinery, including Dicer and Drosha to stress granules. Consequently, miRNA production is compromised, with several miRNAs (i.e. *miR-17~92* and *miR-218*) perhaps directly participating in ALS disease onset and progression [56, 142]. Mislocalization of ALS-related proteins such as FUS and TDP-43 in the cytosol rather than nucleus of MNs has been observed in ALS patients, but the mechanism remains unclear [125, 146].

A recent study highlighted differences in RNA concentration between the nucleus and cytosol. In the nucleus where the concentration of RNA is high, ALS related-proteins such as TDP-43 and FUS are soluble, but protein aggregations form in the cytosol where the concentration of RNA is low, suggesting that RNA could serve as a buffer to prevent LLPS [84]. Collectively, these findings indicate that not only are RNAs the binding blocks for RBPs, but may also serve as a solvent to buffer RBPs and prevent LLPS. Accordingly, persistent phase separation under stress conditions could enhance formation of irreversible toxic aggregates of insoluble solidified oligomers to induce neuronal degeneration [148]. Although many neurodegenerative diseases have been associated with RNP granules, and primarily stress granules, it remains to be verified if stress granules/LLPS are causative disease factors *in vivo*. Many other questions remain to be answered. For instance, are the lncRNAs/RNPs mentioned above actively involved in RNP granule formation? Given that purified cellular RNA can self-assemble *in vitro* to form assemblies that closely recapitulate the transcriptome of stress granules and the stress granule transcriptome is dominated by lncRNAs [63, 144], it is likely that the RNA-RNA interactions mediated by abundantly expressed lncRNAs might participate in stress granule formation in ALS contexts. Similarly, do prevalent RNA modification and editing events in lncRNAs [159] change their hydrophobic or charged residues to affect LLPS and the formation of RNP granules to give rise to disease pathologies? It will be tantalizing to investigate these topics in the coming years.

## Conclusion and perspective

Over the past decade, increasing evidence has challenged the central dogma of molecular biology that RNA serves solely as a temporary template between interpreting genetic information and generating functional proteins [23]. Although our understanding of lncRNAs under physiological conditions is increasing, it remains to be established if all expressed lncRNAs play particular and functional roles during embryonic development and in disease contexts. Versatile genetic strategies, including CRISPR-Cas9 technology, have allowed us to clarify the roles of lncRNA, the individual lncRNA transcripts *per se*, and their specific sequence elements and motifs [42]. Taking spinal MN development and degeneration as a paradigm, we have utilized ESC-derived MNs and patient iPSC-derived MNs to dissect the important roles of lncRNAs during MN development and the progression of MN-related diseases such as ALS and SMA. A systematic effort to generate MN-hallmark lncRNA knockout mice is underway, and we believe that this approach will help us understand the mechanisms underlying lncRNA activity, paving the way to develop new therapeutic strategies for treating MN-related diseases.

## Data Availability

Not applicable.

## Abbreviations

AD: Alzheimer’s disease
ALS: Amyotrophic lateral sclerosis
ASO: Antisense oligonucleotides
ATXN2-AS: Ataxin 2 antisense transcript
BACE: β-secretase-1
C9ORF72: Chromosome 9 open reading frame 72
CTCF: CCCTC-binding factor
CNS: Central nervous system
ESC: Embryonic stem cell
fALS: Familial amyotrophic lateral sclerosis
Foxp1: Forkhead box protein P1
FTD: Frontotemporal dementia
fFTD: Familial frontotemporal dementia
FTLD: Frontotemporal lobar degeneration
FUS/TLS: Fused in sarcoma/translocated in sarcoma
*hsrω*: *Heat shock RNA ω*
Hox: Homeobox
iPSC: Induced pluripotent stem cell
LLPS: Liquid-liquid phase separation
lncRNA: Long non-coding RNA
Meg3: Maternally expressed gene 3
miRNA: microRNA
MN: Motor neuron
Mnx1: Motor neuron and pancreas homeobox 1
NEAT1: Nuclear enriched abundant transcript 1
ncRNA: Non-coding RNA
nt: Nucleotide
pMN: Motor neuron progenitor
PRC2: Polycomb repressive complex 2
RA: Retinoic acid
RBP: RNA-binding protein
RNP: Ribonucleoprotein
sALS: Sporadic amyotrophic lateral sclerosis
Shh: Sonic hedgehog
SMA: Spinal muscular atrophy
SMN: Survival motor neuron
TDP-43: TAR DNA-binding protein 43
Uchl1: Ubiquitin carboxyterminal hydrolase L1
UTR: Untranslated region
Xist: X-inactive specific transcript
